# Cardiac Tissue Engineering for Translational Cardiology: From In Vitro Models to Regenerative Therapies

**DOI:** 10.3390/bioengineering12050518

**Published:** 2025-05-14

**Authors:** Abdullah Jabri, Bader Taftafa, Abdulaziz Mhannayeh, Mohamed Alsharif, Tasnim Abbad, Sana Ahmed, Eman A. Alshehri, Abdulrahman Elsalti, Jibran Khan, Tanveer Ahmad Mir, Ahmed Yaqinuddin

**Affiliations:** 1 College of Medicine, Alfaisal University, Riyadh 11533, Saudi Arabia; ajabri@alfaisal.edu (A.J.); betaftafa@alfaisal.edu (B.T.); amohanaya@alfaisal.edu (A.M.); moalsharif@alfaisal.edu (M.A.); tabbad@alfaisal.edu (T.A.); jibrar@alfaisal.edu (J.K.); 2 Tissue/Organ Bioengineering & BioMEMS Laboratory, Organ Transplant Centre of Excellence (TR&I-Dpt), King Faisal Specialist Hospital and Research Centre, Riyadh 11211, Saudi Arabia; sana.ahmed32@gmail.com (S.A.); aealshehri@kfshrc.edu.sa (E.A.A.); 3 International School of Medicine, Istanbul Medipol University, Istanbul 34810, Turkey; abdulrahman.el@std.medipol.edu.tr

**Keywords:** bioengineering, cardiovascular system, in vitro models, disease modeling, regenerative medicine

## Abstract

Cardiovascular diseases (CVD) are the primary cause of death and disability around the world. Over the past decades, several conventional model systems based on two-dimensional (3D) monolayer cultures or experimental animals have been adopted to dissect and understand heart diseases in order to develop treatment modalities. However, traditional models exhibit several limitations in recapitulating human-specific key physiological and pathological characteristics, which highlights the necessity of developing physiologically relevant models. In recent years, tissue engineering approaches have been extensively employed to generate revolutionary three-dimensional (3D) cardiac models. In particular, the combined use of various bioengineering strategies and cellular reprogramming approaches has facilitated the development of various models. This review presents an overview of different approaches (bioprinting, scaffolding, and electrospinning) for creating bioengineered cardiac tissue models. Next, a broad survey of recent research related to the modeling of various cardiac diseases is presented. Finally, current challenges and future directions are proposed to foster further developments in the field of cardiac tissue engineering.

## 1. Introduction

Heart disease is one of the deadliest afflictions, with high mortality rates worldwide. Cardiovascular diseases (CVDs), such as ischemic heart disease and cardiomyopathy, are among the leading causes of global mortality, with rising death rates and an increasing healthcare burden. Treating heart failure often requires a combination of lifestyle changes, medications such as diuretics, medical devices like pacemakers and left ventricular assist devices (LVADs), or surgical interventions [1]. However, these methods are associated with various challenges, risks, high costs, and multiple limitations. Therefore, investing more in cardiac research is crucial, as it plays a critical role in preventing and treating CVDs, which remain a leading cause of morbidity across the globe. As cardiovascular diseases continue to pose a global health burden, sustained investment and collaboration in cardiac research are essential for advancing medical innovation, reducing healthcare costs, and, ultimately saving lives [2].

**Figure 1:**
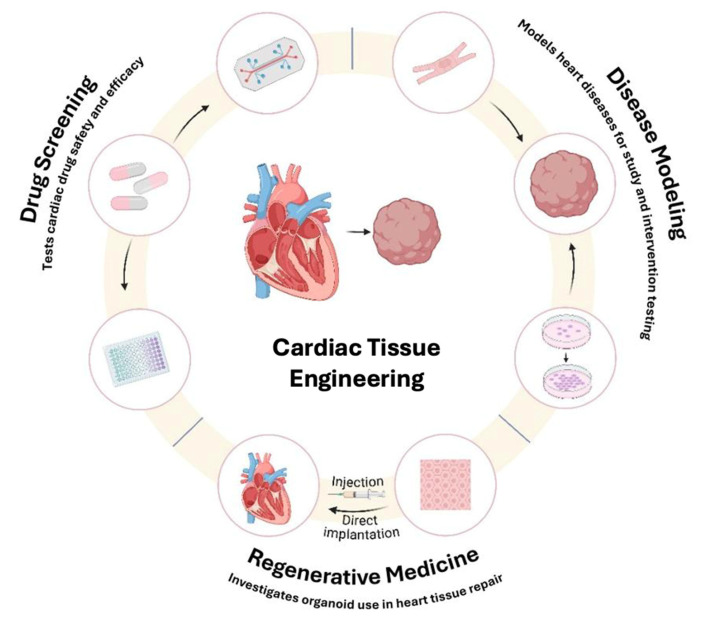
This figure illustrates the key applications of cardiac tissue engineering, including disease modeling, regenerative medicine and drug screening. It also highlights the interconnection between these domains, where advancements in one area enhance the others, positioning cardiac tissue engineering as vital in cardiovascular research and therapeutic development.

In the search for treatments for CVDs through research, tissue models, free from the ethical challenges commonly associated with traditional animal models, also address disparities between species. Traditional models, such as animal testing and 2D cell cultures, have limitations in cardiac research, as many animal models fail to replicate human heart conditions, and 2D cell cultures lack the complexity required to accurately mimic the human heart. Unfortunately, both models lack the physiological factors necessary for accurate assessment of cardiac function [3]. Innovative regenerative therapies offer promising solutions for repairing and replacing damaged cardiac tissue. Tissue engineering is an advancing field that holds significant potential for cardiac tissue regeneration and repair, making it a promising strategy to overcome this global crisis. Tissue engineering is based on three core elements: matrices, living cells, and cytokines, providing multiple approaches for their application in stem cell engineering and the development of biomimetic scaffolds. It holds the promise of reducing the essential need for organ grafting and minimizing medication side effects. Although still in its early stages of development, recent breakthroughs highlight to pioneer a new era in disease treatment [4]. Advanced bio-functional biomaterials and tissue-engineered heart models have demonstrated to be effective reinforcement materials for understanding heart function, improving diagnostics, and developing targeted therapies (Figure 1).

**Figure 2:**
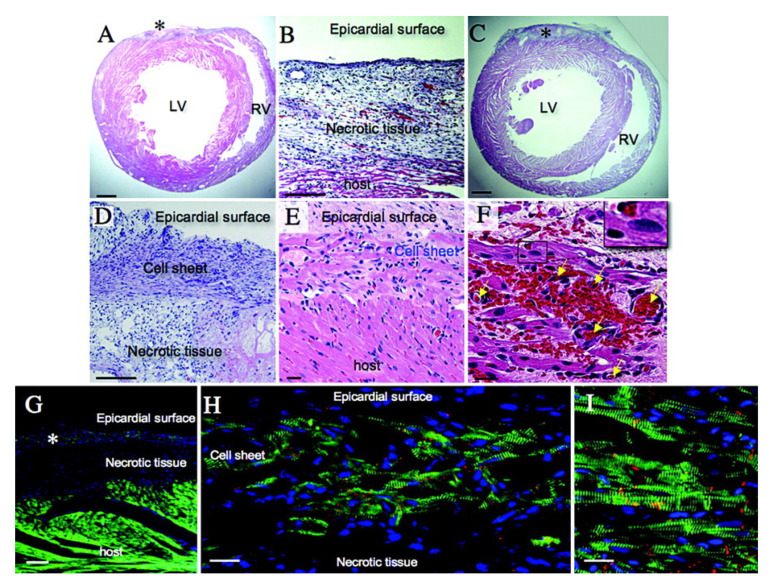
Histological analysis of transplanted cardiomyocyte sheets in the infarcted myocardium. (**A**,**B**) A section of the heart obtained from the sMI group was stained with hematoxylin and eosin. Myocardial necrotic areas 0.15 to 0.3 mm in depth at the epicardial surface are illustrated by hematoxylin staining (*). (**C**–**E**) Image of a sample from the sMI+ sheet group. On the epicardial surface of the sMI area, at a depth of 0.1 to 0.2 mm, an eosin-like staining layer, the grafted cell sheet, can be seen (*). The grafted cell sheet is directly attached to the host heart at the margin of the sMI area (**E**). (**F**), Microvessels are apparent in the grafted myocardial cell sheets (yellow arrows). (**G**–**I**) Laser confocal microscopic view of the grafted cell sheet and host heart, triple-stained with anti–α-actinin (green), anti–connexin 43 (red), and DAPI (blue). ((**G**), α-actinin–positive cell sheet at the epicardial surface of the necrotic tissue (*). ((**H**) Clear striation pattern of α-actinin and diffuse connexin 43 staining in the cell sheet. (**I**) Host image below the necrotic area. Scale bars: 1 mm (**A**,**C**); 100 μm (**B**,**D**,**G**); 20 μm (**E**,**F**,**H**,**I**). Adapted from [6], with copyright permission under the terms of the CC-BY 4.0 license.

Histological studies of grafted cardiac cell sheets have shown the presence of functional cardiomyocytes with clear α-actinin striations and microvascular development, demonstrating integration with the host myocardium and survival on the epicardial surface after transplantation (Figure 2). Furthermore, stem cells, scaffolds, and 3D printing have been used to develop cardiac structures, such as artificial hearts, and build tissue constructs in vitro for drug screening [4,5]. By examining the application, challenges, and potential future developments of these scaffolds, we provide a comprehensive overview of the current landscape in this field.

## 2. Structural and Functional Characteristics of the Human Heart

The human heart is composed primarily of cardiomyocytes, which make up about 25% to 30% of its cellular content, with the majority of the remaining cells being non-myocyte cells [7]. These include approximately 64% endothelial cells, about 9% leukocytes, and roughly 27% resident mesenchymal cells, predominantly fibroblasts. Additionally, populations of vascular smooth muscle cells, pericytes, epicardial cells, conductance cells, neurons, immune cells, and other less common cells play significant roles in heart regeneration and fibrotic remodeling [8,9,10]

Cardiomyocytes are diverse and include sinoatrial nodal cells, atrioventricular nodal cells, atrial cells, ventricular cells, and Purkinje cells, all of which function in conjunction with the vascular system and various non-myocyte cell types [11]. Cell-cell interactions are essential as non-myocytes and cardiomyocytes are functionally interlinked, playing a significant role in both physiology and pathophysiology.

Cardiac cells are integrated into a complex three-dimensional structure known as the extracellular matrix (ECM), which consists of non-cellular components [12]. In addition to the crucial cell-cell interactions within a restricted geometry, interactions between cells and the ECM are fundamental for achieving cellular polarity and cell-cell connections along the axial direction, which are necessary for mechanical and electrical linking [13]. The core structural component of the cardiac extracellular matrix (ECM) is predominantly composed of collagen, supplemented by laminin, fibronectin, fibrillin, and elastin. These elements are perpetually produced by various cell types and come together to form a meticulously arranged framework. Additionally, the ECM’s non-structural component contains a wide range of proteins, crucial for its plasticity. These proteins are organized into three principal categories: glycoproteins, proteoglycans, and glycosaminoglycans [14,15].

## 3. Methods for Generating Cardiac Tissue-Engineered Models In Vitro

**Figure 3:**
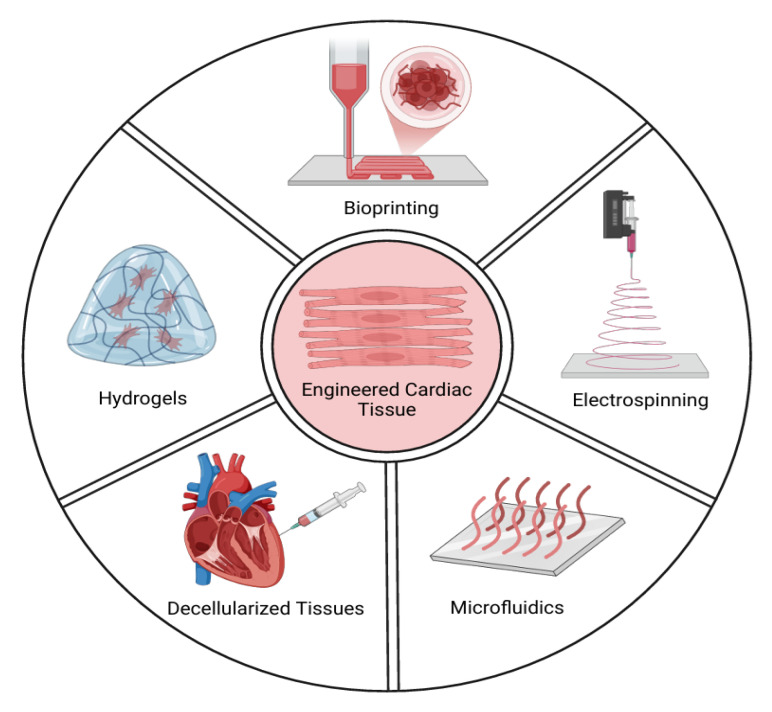
Key technologies and approaches in tissue engineering and regenerative medicine. The figure highlights five critical methodologies: hydrogels, bioprinting, electrospinning, decellularized tissues, and microfluidics. These techniques represent advancements in the creation of functional tissues for medical applications, ranging from scaffold fabrication to complex tissue modeling.

Different cardiac cell types, including cardiomyocytes (CMs), endothelial cells (ECs), and cardiac fibroblasts (CFBs), are commonly found in cardiac 3D models [16]. There are two strategies for producing in vitro cardiac organoids: scaffold-based and scaffold-free methods. While scaffold-free techniques generally involve encouraging the spherical aggregation of cultured cells in an anti-adhesive environment, scaffold-based methods use biomaterials such as hydrogels or decellularized bioscaffolds. Additionally, some recently developed techniques, such as microarray technology, 3D bioprinted models, and scaffolds based on electrospun fiber mats, have been used to facilitate the formation of cardiac organoids (Figure 3). Scaffold-free 3D cultures are created by the self-assembly of cells, while scaffold-based cultures use hydrogels or other scaffolds to support tissue replicas. In both cases, the cells are organized into a three-dimensional structure, which is essential for preserving their morphology, phenotype, and polarity. Scaffold-free systems have the ability to generate artificial 3D heart tissues that preserve their mechanical integrity without requiring external support [17].

### 3.1. Scaffold-Free Systems

The creation of cardiac organoids using scaffold-free techniques largely relies on the self-assembly properties of cells derived from pluripotent stem cells (PSCs). Initially, 3D structures are generated in suspension cultures on low-attachment plates, which incorporate either purified induced cardiomyocytes (iCMs) or iCMs directly differentiated from embryoid bodies [18,19]. In the natural heart, cardiomyocytes (CMs) receive vital signals from endothelial cells (ECs) and cardiac fibroblasts (CFs) to develop into a cell type capable of enhanced calcium handling and contractility. ECs supply CMs with oxygen and nutrients and secrete paracrine factors, such as nitric oxide and endothelin-1, which regulate CM contraction and prevent apoptosis. CFs, situated between the cardiac muscle layers, provide structural support and assist in cardiac conduction. They also release fibroblast growth factor, prompting ECs to produce vascular endothelial growth factor, thereby stimulating angiogenesis. The interaction between CMs, ECs, and CFs results in improved tissue organization in organoids, which enhances CM function and EC-mediated angiogenesis [20,21,22]. As a result, creating a cardiac organoid using a mix of primary or pluripotent stem cell-derived CMs, ECs, and CFs results in a more physiologically accurate microtissue compared to organoids composed only of CMs [23]. Various heart cell types are systematically developed from embryoid bodies in a step-by-step manner. Researchers highly prefer mosaic cardiac organoids, which contain most heart cell types [24,25]. However, these organoids face challenges, such as inconsistency in reproducibility and a lack of full complexity, as they still do not replicate all the cell types and features found in native heart tissue, such as perfusable blood vessels, four-chamber structures, the cardiac conduction system, and resident immune cells [23].

In a study by Pretorius et al., a layer-by-layer technique was used to create an in vitro heart tissue model without a scaffold [26]. This method involves combining human-induced pluripotent stem cell-derived cardiomyocytes (hiPSC-CMs) with a fibrin matrix, which is placed in a polycarbonate frame to form the first layer. A second layer of endothelial cells (ECs) was added on top, followed by a third layer of cardiac fibroblasts (CFs). The entire tissue was placed on polydimethylsiloxane supports, allowing it to be immersed in fresh medium. Immunofluorescent analysis has revealed successful cell migration, rearrangement, and stable gene expression, emphasizing the significance of co-culturing various heart cell types. Another scaffold-free method uses magnetic forces, wherein cells are incubated with magnetic nanoparticles that adhere to their membrane. These cells are placed in non-adhesive plates and manipulated with a magnet to levitate toward the air-liquid interface, forming a 3D structure [27]. Additionally, the hanging drop method involves positioning cell suspension droplets in Petri dish lids, where gravity and surface tension cause the cells to self-aggregate and form 3D tissues. Beauchamp et al. used this method to develop 3D heart tissue by co-culturing hiPSC-CMs with other cardiac cell types [28]. After confirming that the tissue was beating, the cell aggregates were placed in a mold to promote tissue compaction and maturation. This approach resulted in a uniform cardiac patch with synchronized beating. However, self-aggregation methods face limitations due to the less mature state of cardiomyocytes compared to those in the adult heart, and challenges arise in managing the layering of cells [29,30].

### 3.2. Scaffold-Based Systems

Certain support structures are necessary for scaffold-based systems because they guide the alignment of cardiomyocytes (CMs), which is essential for their maturation. Scaffolds are porous structures that facilitate the transport of nutrients, growth stimulants, and gas exchange. They can be constructed using natural macromolecules or synthetic materials. They facilitate differentiation, migration, adhesion, and proliferation of cells [17,31]. and Because matrigel closely resembles the original extracellular matrix (ECM), it is frequently employed in 3D cultures to cultivate a variety of epithelial and endothelial cells. However, its complicated composition, batch unpredictability, inconsistent cell culture experiment results, and inability to be adjusted for certain organoid niches hinder its use in organoid technology. Consequently, there has been a shift toward exploring other well-defined natural and synthetic scaffolds. Natural polymer-based hydrogels like hyaluronic acid, alginate, chitosan, and polyethylene glycol (PEG), are increasingly preferred for their chemical and mechanical properties that closely mirror the human ECM [32,33,34]. In addition, these synthetic polymers are becoming increasingly popular for cardiac tissue engineering because of their customizable and biocompatible qualities. The design and functionality of scaffolds are significantly impacted by the solubility properties of these materials. Because of their high water solubility, hyaluronic acid and alginate can quickly form hydrogels in physiological settings, which makes them ideal for injectable cardiac patches or minimally invasive regenerative scaffold delivery [35,36]. Although it is mostly soluble in mildly acidic solutions, chitosan can form bioadhesive and mechanically robust structures that are appropriate for applications that need long-term in situ support, like bioactive stent coatings or epicardial patches [37,38]. Through chemical modification, PEG-based materials provide adjustable solubility and degradation kinetics, making them attractive options for dynamic scaffolds in myocardial tissue repair and controlled drug delivery systems [39]. The engineering of cardiac constructs suitable for particular clinical interventions, such as myocardial regeneration or therapeutic agent delivery after myocardial infarction, is made possible by the strategic selection of scaffold materials based on their mechanical and solubility characteristics. Scaffolds can be customized using a variety of materials with certain qualities based on the objectives of the experiment. Miller et al. used a 3D hydrogel scaffold made with a micro-continuous optical printing method to build a microtissue [31]. This approach uses UV light to polymerize a pre-polymer solution that contains gelatin methacrylate (GelMA) and cells (hiPSC-CMs and CFs). The scaffold lines were printed using this solution onto a layer of GelMA pillars that had been marked with fluorescent beads. The fluorescently indicated pillar layer moved, indicating that the tissue underwent beating contractions within a few days. By manipulating the GelMA concentration and UV exposure duration, the researchers were able to regulate the stiffness of the hydrogel by modifying the hydrogel’s pore size. Sarcomere staining revealed significant cell alignment, and gene expression analysis showed increased expression of maturity markers in hiPSC-derived cardiomyocytes (hiPSC-CMs), including CACNA1C, RYR2, MYH6, MYH7, MLC2C, and TNNT genes. Some researchers have proposed using aligned coaxial nanofibers as scaffolds to mimic the heart’s extracellular matrix (ECM), allowing cardiomyocytes to align with the nanofibers and adopt an elongated shape similar to adult CMs in vivo [40,41]. Ahn et al. created nanofiber scaffolds from PCL and dopamine hydrochloride, functionalized with polydopamine (PDA) to improve cell adhesion. The system also included sensors to monitor cell contractility. Neonatal rat cardiomyocytes cultured on these scaffolds showed proper alignment, sarcomere Z-line density, and length, confirming CM maturation. Additionally, using a Ca2+-sensitive dye demonstrated synchronous calcium transients, indicating the scaffold’s ability to support the development of functional, contractile tissue [42]. However, scaffold-based systems are associated with challenges, including high costs, the need for specialized technical expertise, and lengthy preparation times [29]. An additional challenge, often underrepresented in cardiac tissue engineering, is the host immune response to the scaffold materials. The relationship between host immune system cells and implanted materials is crucial in scaffold-based cardiac tissue engineering. Scaffolds can alter immune responses by affecting fibrotic tissue formation, cytokine secretion, and macrophage polarization [43,44]. The particular type and degree of immune activation are strongly influenced by material characteristics like porosity, stiffness, and surface chemistry. Constructive cardiac remodeling and functional tissue integration depend on the design of scaffolds that minimize pro-inflammatory (M1-like) responses and enhance anti-inflammatory (M2-like) macrophage phenotypes [45]. More physiologically relevant platforms for assessing regenerative therapies might be offered by future cardiac tissue models that incorporate immune cell interactions.

### 3.3. Cardiac Cell Culture in Decellularized Hearts

**Figure 4:**
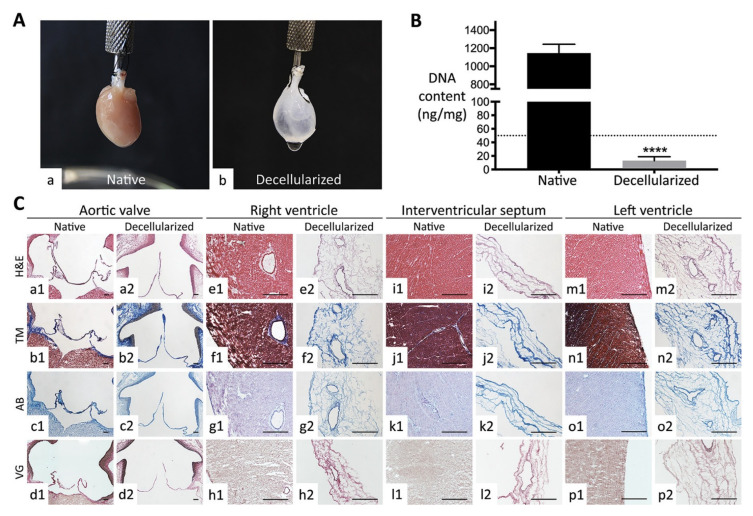
Whole rat hearts before and after decellularization procedure. (**A**) Macroscopic images of (**a**) native and (**b**) decellularized organs. (**B**) Validation of decellularization by quantifying DNA content in native and decellularized samples. **** *p* < 0.0001. (**C**) Validation of decellularization by histological evaluation of organ architecture in native and decellularized states using Haematoxylin−Eosin (H&E), Masson’s trichrome (MT), Alcian blue (AB), and elastic Van Gieson (VG) staining. The valve apparatus, including the aortic valve, right and left ventricles, and interventricular septum of decellularized hearts, showed the preservation of the extracellular matrix in decellularized samples. (**a1**–**p2**) Representative histological images of the aortic valve (**a1**–**d2**), right ventricle (**e1**–**h2**), interventricular septum (**i1**–**l2**), and left ventricle (**m1**–**p2**) stained with H&E (rows 1), MT (rows 2), AB (rows 3), and VG (rows 4) for native (odd-numbered panels) and decellularized (even-numbered panels) tissues. Adapted from [47] with copyright permission under the terms of the CC-BY-NC-ND 4.0 license.

In order to construct a layer of beating cardiac tissue, the conventional approach to heart tissue engineering is to integrate beating cardiomyocytes (CM) and other cardiac cells into biomaterials. The majority of cardiac tissues are created using rat neonatal CMs [46]. Furthermore, most scaffolds lack conserved three-dimensional architectures that aid in reconstructing vascular and muscle structures. Decellularized whole hearts provide the intact cardiac scaffolds required to create 3D heart tissues or bioartificial hearts because they maintain the local niches and natural matrix components [47,48]. Due to poor CM differentiation efficiency, earlier attempts to repopulate decellularized mouse hearts with undifferentiated human embryonic stem (ES) cells failed to produce functioning heart structures [49]. Lu et al. used human iPS cell-derived multipotential cardiovascular progenitors (MCPs) to repopulate intact decellularized mouse hearts, successfully creating functional human heart tissues. This work presents the first application of cardiac tissue engineering using human cardiovascular progenitor cells. Similarly, Guyette et al. used human iPSC-derived CMs to repopulate decellularized human whole heart (WH) and decellularized human cardiac slices (200 µm thick). In biomimetic growing conditions, seeded dECM scaffolds demonstrated both electrical conductivity and metabolic function, and they produced force-generating cardiac tissue with spontaneous contraction [50]. Another study demonstrated, for the first time, a human-sized heart scaffold that had been repopulated with cardiac cells produced from hiPSCs that displayed native-like physiology, architecture, and drug responsiveness [48]. Collectively, these studies demonstrate the significant potential of cardiac decellularized extracellular matrix as a suitable base for cardiac tissue engineering applications (Figure 4).

### 3.4. Microfluidics Technology

**Figure 5:**
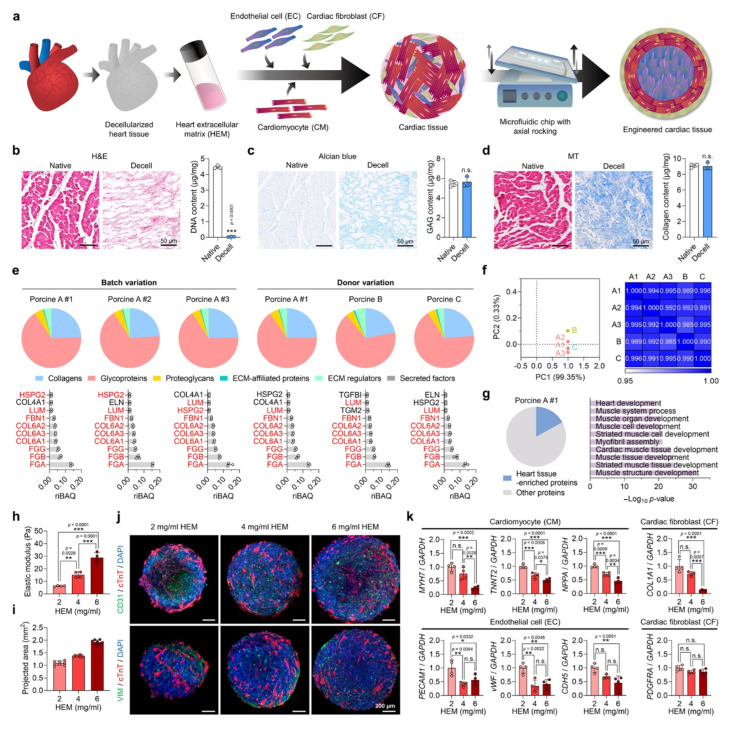
(**a**) Schematic representation of human cardiac tissue engineering using different cell types, heart-derived acellular matrix-hydrogel, and microfluidic platform. (**b**) Confirmation of decellularization using hematoxylin and eosin staining and analysis of DNA content, (**c**) Characterization of decellularized matrix using Alcian blue staining to analyze glycosaminoglycan (GAG) content, and (**d**) Masson’s trichrome (MT) staining to analyze collagen content in native porcine heart tissues and after decellularization. (**e**–**g**) Proteomic analysis of acellular extracellular matrix derived from different batches. (**e**) (A#1, A#2, and A#3) and different donors (A, B, and C). (**f**) Total protein analysis in different acellular extracellular matrix samples (A#1, A#2, A#3, B, and C) using principal component analysis and Pearson’s correlation analysis methods. (**g**) Percentage of heart tissue-enriched proteins among total proteins in acellular extracellular matrix sample A#1 (**left**) and enriched proteins analyzed by Gene Ontology Biological Processes (**right**). (**h**) Elastic moduli of acellular extracellular matrix-derived hydrogels prepared in a concentration-dependent manner (2, 4, and 6 mg/mL). (**i**) Cardiac tissues generated using hydrogels with different concentrations of acellular extracellular matrix (4 and 6 mg/mL). (**j**) Immunofluorescent images showing the expression of different surface markers in each group of cardiac tissues containing cardiomyocyte (CM; cTnT), cardiac fibroblast (CF; VIM), and endothelial cells (EC; CD31). (**k**) Relative mRNA expression patterns for cardiomyocytes (TNNT2, MYH7, and NPPA), endothelial cells (vWF, PECAM1, and CDH5), and cardiac fibroblasts (COL1A1 and PDGFRA) in each group. * *p* < 0.05, ** *p* < 0.01, *** *p* < 0.001, n.s. = not significant. Adapted from ref. [51], with copyright permission under the terms of the CC-BY-NC-ND 4.0 license.

Microfluidic technology enables the fabrication of more-relevant disease models to better decipher cardiac function [51] (Figure 5). Microfluidic chips employ polydimethylsiloxane substrates to create uniform and reproducible tissue constructs. These platforms simulate tissue structure and function at the micron level and are widely used in various applications, including drug screening systems, regenerative medicine, and cancer biology [52]. One innovative use of this methodology was a tissue-cultivation platform designed to be independent of the cell source and suitable for drug testing under electrical pacing, featuring a plastic structure that supports the online noninvasive tracking of key cardiac functions, including passive tension, active force, contractile dynamics, and Ca2+ transients. It also allows for detailed endpoint evaluations of the action potentials and conduction velocity. Through the use of directed cell differentiation paired with electrical field conditioning, this platform enables the creation of electrophysiologically distinct atrial and ventricular tissues that exhibit specific drug reactions and gene expression patterns. Notably, this is the first time that heteropolar cardiac tissues with distinct atrial and ventricular ends have been developed, demonstrating targeted responses to drugs such as serotonin and ranolazine. Importantly, the platform includes an array of microwells on polystyrene sheets secured at both ends with flexible wires made from poly (octamethylene maleate (anhydride) citrate) (POMaC) polymer using adhesive glue. Within these microwells, myocardial tissues are crafted by combining cardiomyocytes, either ventricular, atrial, or both, with cardiac fibroblasts, all embedded in the hydrogel [53].

### 3.5. Bioprinting Technology

**Figure 6:**
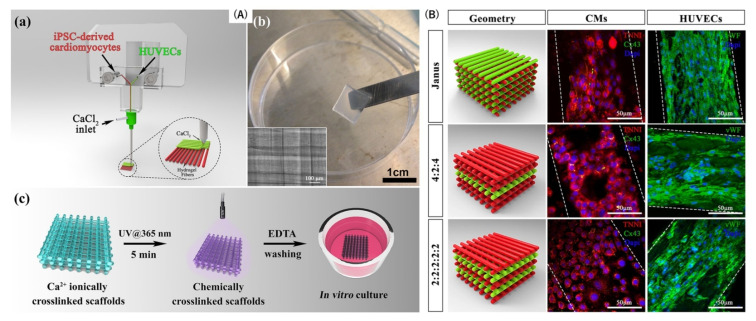
(**A**,**B**): (**a**) Schematic illustration of the bioprinting head system coupled to a coaxial nozzle extruder utilized to print iPSC-derived cardiomyocytes and human umbilical vein endothelial cells (HUVEC). (**b**) Bioprinted construct with a thickness of 10-layers. (**c**) Diagram depicting the fabrication procedure of PEG-fibrinogen-based scaffolds covalently crosslinked to generate bioprinted multicellular structures. (**B**) Representative images of printed cellular structures on day 7 of culture in three different spatial geometries displaying the expression of cardiac biomarkers, including troponin-I (red) and connexin 43 (green) for cardiomyocytes and von Willebrand (green) labeling for HUVEC cells. Adapted from ref. [54], with copyright permission under the terms of the CC-BY-NC-ND 4.0 license.

In order to construct engineered structures based on complex 3D designs that mimic human tissues, 3D bioprinted models employ additive manufacturing technology, which blends biomaterial scaffolds, cells, and growth factor tissues [54] (Figure 6). These multicellular systems are used to study human diseases and developmental biology by simulating an in vivo environment [55]. A 3D cardiac tissue model was recently presented in a study that may improve biomarker analysis and aid in the creation of focused treatment plans for heart disease, thus expanding the knowledge of cardiac biology. For future research on drug cytotoxicity effects, this 3D cardiac cell spheroidal droplet model is expected to offer a platform for promoting heterocellular interactions between cardiac myocytes and fibroblasts. The study produced 3D spheroidal droplets at high throughput after standardizing the 3D bioprinting parameters. These droplets exhibit interconnected porosity, promoting cell survival and functionality [56]. In another study, endothelial cells, fibroblasts, and cardiomyocytes produced from induced pluripotent stem cells (iCells) were combined to create heart tissue. Using a Bio-3D printer, these parts were assembled into tubular structures and positioned on a needle array. While still on the array, the fusion of spheroids and their beat rates were continuously observed. The fusion of spheroids and their beat rate were closely monitored while they were still on the array. Once removed, the constructs underwent electrical stimulation, during which they displayed an increase in beat rate, returning to normal post-stimulation. Histological studies have also shown that cellular reorganization within these constructs resembles the changes observed during organ transplantation. Overall, these findings suggest that tubular cardiac constructs produced via an optimized Bio-3D printing method could potentially be utilized in clinical settings and for drug testing, offering solutions for the scarcity of donor tissues and issues with transplant rejection. This represents the first instance of using such a technique to produce and validate cardiac tubular constructs as functional cardiac pumps [57].

Most 3D bioprinted tissue substitutes are generally constrained to small-sized structures, a limited number of cell types (typically one or two), and relatively straightforward structures with restricted biofunctionality [58].

### 3.6. Electrospinning Technology

Electrospinning is an adaptable and straightforward method for creating nanofibers with customizable structures, properties, and functions. This technique uses a range of natural and synthetic biodegradable polymers that are suitable for crafting cardiac organoids. A jet of charged polymer is placed on a grounded collector during the electrospinning process. The resulting fiber mats have a random orientation if the collector stays stationary, but aligned nanofibers are produced when it spins rapidly [59]. Cell migration, proliferation, and survival can all be improved by changing the nano-architecture of scaffolds by adjusting the fibers’ diameters. Through the replication of microenvironmental structures such as the extracellular matrix (ECM), this modification also promotes mechanical properties [55]. In order to improve the material and biological performance of electrospun scaffolds, a combinatorial approach combining natural and synthetic polymers has been investigated. Facilitating enhanced cell adhesion, survival, development, migration, and deeper cell penetration into the scaffold’s core is the main goal of these polymer mix systems [60]. Three different types of electrospun scaffolds were created for tissue engineering applications in a study by Nagiah et al.: coaxial scaffolds with a PCL core and an f-gelatin sheath, f-gelatin alone, and f-gelatin and PCL combined in a 1:1 ratio [61]. The results demonstrate a straightforward process for creating hybrid biodegradable nanofibrous scaffolds that can cross-link when exposed to visible light. These scaffolds can be used to create cardiac tissue-engineered models in an efficient manner.

### 3.7. Clinical Relevance of Engineered Cardiac Tissues

Engineered cardiac tissues are important models for simulating pathological remodeling processes following myocardial infarction (MI). Fibrotic scar formation, altered extracellular matrix (ECM) composition, impaired contractility, and disrupted vascularization are among the complex biomechanical and biochemical changes that occur in the heart after MI [22,62]. Scaffold-free and scaffold-based engineered constructs allow researchers to mimic these processes by organizing cardiomyocytes (CMs), fibroblasts, and endothelial cells (ECs) into three-dimensional architectures that resemble post-injury tissue microenvironments. Specifically, fibrosis-driven mechanical stiffening is replicated by scaffold-free organoids that incorporate CFs, and the mechanical heterogeneity of the infarcted myocardium is modeled using engineered hydrogels with adjustable stiffness [63,64]. The study of human cardiac tissue remodeling under pathophysiologically relevant mechanical loads and ECM compositions is further facilitated by decellularized heart matrices [48,50]. Microfluidic devices facilitate the modeling of ischemic gradients, mechanical stress, and inflammatory environments typically associated with heart failure [53]. Moreover, the immunological response to implanted materials, especially macrophage phenotype modulation, plays a central role in determining whether engineered cardiac tissues promote healing or exacerbate fibrotic outcomes [45]. Collectively, these in vitro systems provide a critical platform for preclinical drug screening, regenerative strategy development, and mechanistic studies on cardiovascular disease progression.

## 4. 3D Modeling Techniques in Cardiac Tissue Engineering

In vivo, cardiac tissues are enmeshed in extracellular matrix (ECM) proteins and exposed to a wide range of biochemical, mechanical, electrical, and other stimuli that cause appropriate reactions and precisely calibrated changes in gene expression [65]. Engineering three-dimensional myocardial tissues offer significant benefits for primary cardiomyocyte (CM) cultures used to explore the biology, physiology, and pharmacology of the human heart. Unlike traditional flat cultures, CMs maintained in a three-dimensional setting demonstrate extended survival and preservation of their ability to contract. Furthermore, combining CMs with other cell types, such as endothelial cells and cardiac fibroblasts, enhances integration and functional outcomes in myocardial infarction models within living organisms, showing superior results compared to the use of CM sheets alone [66]. Current 3D cell model production methods fall into three categories: (1) Hydrogel models, which involve embedding cells in matrices made from ECM proteins like matrigel or collagen, exemplified by cardiac rings and strip models. (2) Rigid scaffold models, in which cells are grown on or within materials like polyurethane, shaped into inserts, beads, or sponges, such as cardiac patches. (3) Scaffold-free techniques, which include leveraging cell self-organization into structures like spheroids and cell sheets, without external support. These classifications encompass a range of 3D culture environments, from hydrogels to scaffold-based and scaffold-free systems [67].

### 4.1. Spheroids

Spheroids are spherical clusters of multiple cells. They can be generated through various techniques: (1) by allowing floating cells to self-assemble on non-stick surfaces or within spinning vessels, leading to spheroids of varying dimensions; (2) by using the hanging drop technique for consistent spheroid size; or (3) by employing microfluidic technology [67]. The size of these spheroids is typically limited to a few hundred nanometers due to the limits of diffusion, ensuring that oxygen and essential nutrients reach the core [68]. In addition to cardiotoxic and pro-arrhythmic pharmacological effects, cardiac spheroids are characterized by spontaneous contraction, and the resulting motion is useful for evaluating spheroid survival [69]. Their significant advantage lies in their capability for rapid and efficient production with limited use of expensive cells, offering a high-speed, low-cost production process [65].

Furthermore, these spheroids facilitate the execution of 3D co-cultures. Giacomelli et al. observed that combining cardiomyocytes generated from induced pluripotent stem cells (iPSCs) with cardiac fibroblasts and endothelial cells provides an environment that more accurately mimics the natural cardiac environment [70]. This tri-cellular setting exhibited notable enhancements in sarcomeric architecture, including T-tubules, as well as improvements in contractility, electrophysiology, and mitochondrial respiration.

They also play a key role in a number of stem cell technologies, such as cell therapy methods, embryo toxicity testing (EST-toxicity assay), and culture as embryoid bodies. Gallet et al. showed that “cardiospheres” that are up to 100 μm in diameter, grown in suspension cultures from biopsy-obtained cells that exhibit both stromal and cardiac stem cell markers, are utilized in both preclinical research and clinical trials [71].

### 4.2. Cell Sheets

Shimizu et al. developed an innovative approach within scaffold-free techniques in which cells are cultured on temperature-responsive culture surfaces [72]. These platforms enable the spontaneous detachment and harvest of entire layers of interconnected cells, along with the extracellular matrix (ECM) proteins theyproduce, without the use of proteolytic enzymes like trypsin or collagenase, which can adversely affect the integrity of cardiomyocytes in culture.

This strategy has been applied to create multi-layered constructs of cardiomyocyte sheets, which can be stacked or rolled to form complex tissue structures. These assemblies have been integrated with perfused vascular networks for tissue engineering purposes, including femoral veins and arteries obtained from animals. Remarkably, it has been demonstrated that these vascularized constructs, after undergoing a phase of development and vascularization using a bioreactor, can be connected to a mechanical perfusion system and eventually reintegrated with the animal’s vascular system, facilitating the development of spontaneously beating cardiac tissue constructs in vivo [73].

This technique could serve as the foundation for developing a complete model of the ventricular wall, including different cell types, perfusion, trophic factors, mechanical function, and cell-cell communication [67].

### 4.3. Strips

Cardiac strips or fibers are made by culturing 3D printed cardiac tissues in rectangular molds, which are then anchored to either two flexible pillars or a wire [74,75]. Typically, cells are combined with a hydrogel matrix and dispensed into a mold, where they undergo polymerization and tighten to create pulsating tissue. This motion causes the pillars or wires to bend with every beat. A key benefit of this design is that it imparts a unidirectional orientation to the tissue, enabling straightforward, non-destructive measurements of the active force. The inaugural experiments with this design utilized cells from neonatal rat hearts, resulting in well-organized and aligned cardiac muscles. The force generated by these muscles was assessed through video analysis by tracking the movement of the pillars. These studies demonstrated that this specific formation permits the evaluation of the reaction of constructs to various substances. They found that the tissues responded in a dose-dependent manner to traditional cardiac medications like chromanol, quinidine, and doxorubicin, showing either a change in heart rate or contractile force [75,76].

### 4.4. Patches

Cardiac patches consist of thin layers of cardiomyocytes combined with various other cell types that may be embedded within a scaffold or linked together to form a coherent structure [77]. A key feature of cardiac patches is their ability to be scaled up to sizes of clinical relevance without compromising their functional capabilities, as evidenced by the construction of patches measuring 4 × 4 cm and 3.5 × 3.4 cm [78,79]. The scalability of cardiac patches not only facilitates their application in cardiac repair but also makes them an effective platform for drug screening. In one study, hiPSC-CMs and cardiac fibroblasts were exposed to ECM proteins and mixed in different ratios with endothelial cells. An optimal ratio of hiPSC-CMs to CFs to ECs allowed for observable changes in contraction properties in response to E-4031 and isoproterenol, indicating the utility of cardiac patches in drug screening applications [80]. Additionally, these patches enable the direct measurement of both active and passive contractile forces and can be subjected to mechanical and electrical stimulation. Unlike cardiac spheroids, cardiac patches provide adjustable levels of structural and functional anisotropy.

### 4.5. Rings

Cardiac rings, a form of 3D engineered cardiac tissue (ECT), are formed by casting in circular molds that feature a central core, facilitating their consolidation around this axis. Post-formation, these rings can be moved onto rods for passive stretching to aid in their development or attached to force sensors to evaluate both the active and passive forces generated by the tissue [79]. The circular design offers several benefits, including a more uniform distribution of force throughout the tissue, a shape that closely mirrors the heart, and the ability to be downsized for use in large-scale screenings. This distinctive structure also allows the study of re-entrant waves, which are key indicators of arrhythmias. Furthermore, research by Li et al. has shown that these re-entrant waves can enhance the growth of circular engineered cardiac tissues [81,82,83]. They observed enhanced structural organization, along with better calcium management and contraction capabilities in cardiomyocytes subjected to high frequencies without the need for electrical stimulation.

## 5. Applications in Disease Modeling, Drug Screening, and Toxicity Testing

### 5.1. Disease Modeling

#### 5.1.1. Congenital Heart Diseases

Congenital heart disease (CHD) is a significant health concern, accounting for approximately one-third of all major congenital anomalies [84]. The complexity and diversity of CHD necessitate extensive research to understand its underlying mechanisms and develop effective treatments. Human-induced pluripotent stem cells (iPSCs) have recently become a significant tool for disease modeling. iPSCs have been used to model inherited cardiac conditions, including long QT syndrome, ventricular tachycardia, and dilated cardiomyopathy, as well as different types of CHD, including bicuspid aortic valve disease (BAVD) and calcific aortic valve disease (CAVD), cardiac septal defects, Hypoplastic Left Heart Syndrome (HLHS), and Tetralogy of Fallot (TOF) [85,86,87,88,89,90,91,92,93,94].

##### HLHS

Hypoplastic Left Heart Syndrome (HLHS) is a single-ventricle malformation in which the left ventricle, mitral valve, aortic valve, and ascending aorta are underdeveloped [95]. Although HLHS has a strong familial tendency, the genetic factors that cause this condition are poorly understood. Research using HLHS-specific iPSC-derived cardiomyocytes showed developmental and functional abnormalities compared to control iPSC lines and embryonic stem cells [92]. These cells had reduced expression of several cardiac mesoderm marker genes, including MESP1, TNNT2, and GJA1, and delayed GATA4 expression during differentiation. Initially, HLHS was thought that HLHS was due to embryonic hemodynamic instability [96]. However, a recent study by Krane et al. suggested that mutations in specific genes may collectively disrupt the gene programs governing the early stages of cardiogenesis in cardiac progenitor cells [91]. These mutations prevent HLHS cardiomyocytes from responding to growth signals and disrupt cardiomyocyte specification and maturation. This leads to premature cell cycle exit and apoptosis, eventually impairing left ventricle development.

##### TOF

Tetralogy of Fallot (TOF), the most common cyanotic congenital heart defect, is a congenital anomaly that results in pulmonary stenosis, an interventricular defect, biventricular aortic origin, and right ventricular hypertrophy [93]. In 2020, Grunert et al. employed iPSC technology in their study to model and study TOF-specific cardiomyocytes in vitro for the first time [94]. They found disease-causing mutations in tumor protein p53 (TP53), which is crucial for smooth muscle cell migration, fibulin 2 (FBLN2), which regulates atrioventricular valvular-septal morphogenesis, and disheveled associated activator of morphogenesis 2 (DAAM2), which mediates myocardial maturation and WNT signaling. In another study, Han et al. created iPSCs from a child with TOF who had a mutation in the T-box transcription factor 1 (TBX1). TBX1 is a gene located on 22q11 and is crucial for neural crest migration and conotruncal development [97]. These patient-specific iPSC lines aid in identifying the genetic mutations that cause TOF and pave the way for exploring gene therapy for TOF. Table 1 summarizes the iPSC-based models used in CHD research.

#### 5.1.2. Ischemic Heart Diseases

Ischemic heart disease is a condition in which the heart is deprived of oxygen due to inadequate blood flow [98]. Since cardiomyocytes have a limited regenerative capacity, developing IHD models to study and explore therapeutic approaches is crucial [99]. Among these models, human-induced pluripotent stem cells differentiated into cardiomyocytes (hiPSC-CMs) offer a rich framework for studying ischemic heart disease. Thus, several studies have targeted hiPSC-CMs in IHD modeling to demonstrate the effects of hypoxia on the electrical and physiological characteristics of hiPSC-CMs [18,100]. For instance, Richards et al. used non-adhesive agarose hydrogel molds to create mixtures of hPSC-CMs and nonmyocytes, forming three-dimensional cardiac organoids (3D hCOs) [101]. The 3D hCOs were then placed in a hypoxic chamber to induce myocardial infarction. This replicates the standard characteristics of myocardial infarction, including metabolic changes, fibrosis, and calcium handling [102].

Other studies have used iPSC models of ischemic heart disease to assess the pharmacological effects of several drugs on the heart [103,104,105]. For instance, Gaballah et al. created an in vitro model of IHD using hiPSC-CMs fostered under hypoxic conditions. They aimed to examine the impact of hypoxia on hiPSC-CMs and to assess the action of levosimendan, a heart failure medication, on IHD. Their findings revealed that hypoxia slowed calcium transients and caused sarcomere disorganization and cardiac arrhythmia. In contrast, levosimendan demonstrated significant antiarrhythmic properties and protected against sarcomere changes induced by hypoxia.

Several studies have explored different procedures that aim to restore blood flow and regenerate the myocardium after ischemic injury using somatic multipoint and pluripotent stem cells [106]. For example, clinical trials have administered stem cells in different ways, including intravenous, intracoronary, and intramyocardial injections [107]. Another promising strategy involves the development of engineered cardiac patches that are locally implanted on the surface of the damaged area. These patches can have different structures, including molded fibrin/collagen-based hydrogels, electrospun polymeric meshes, stacked cell sheets, and 3D printed/bioprinted constructs [108,109,110,111]. Table 2 summarizes the iPSC-based models used in IHD research.

#### 5.1.3. Cardiomyopathies

Cardiomyopathies are a diverse group of myocardial diseases that lead to aberrant cardiac structures and functions [118]. They are typically inherited and are major contributors to heart failure and sudden cardiac death in young individuals. The utilization of hiPSC-Cms is a growing method that aids in studying the mechanisms of inherited cardiomyopathies and testing potential therapies [88,119,120,121]. In 2018, Long et al. synthesized cardiac muscle using cardiomyocytes produced from human fibroblasts and hiPSCs from patients with Duchenne muscular dystrophy (DMD) to stimulate DMD cardiomyopathy [112]. Additionally, cardiomyocytes were used to create cardiac muscle after correcting the DMD mutation using CRISPR/Cas9. Following their repair, dystrophin expression was restored, and the cardiac tissue’s ability to contract mechanically improved. Interestingly, this repair was possible only by correcting 50% of cardiomyocytes. This work sheds light on the modeling of hereditary cardiomyopathy and the functional implications of gene editing at the level of human cardiac tissue. In another study by Marini et al., a 3D model of a DMD cardiac organoid (DMD CO) helped identify five miRNAs that are significantly expressed in the DMD CO but not in the isogenic controls [113]. These findings encourage further investigation of therapeutic applications targeting these miRNAs.

### 5.2. Drug Screening and Toxicity Testing

HiPSCs-based disease models have made significant advancements in drug discovery, allowing patient-specific iPSCs and disease-affected cells to be used for drug screening. A recent study by Fiedler et al. used iPSC-derived cardiomyocytes (iPSC-CMs) and stimulated them with hydrogen peroxide to simulate ischemic injury in vitro. They used short hairpin RNA-mediated gene silencing to validate the target, mitogen-activated protein kinase kinase kinase kinase-4 (MAP4K4) [114]. After a chemical screen, a new small molecule was found to increase the survival of iPSC-CMs afterischemic injury, showing the potential of iPSC-derived cells for tissue-specific high-throughput drug screening.

During the SARS-CoV-2 pandemic, epidemiological studies showed high mortality and morbidity in COVID-19-positive patients with pre-existing cardiovascular disease, leading to cardiac injury or death [122]. Mills et al. used mature human cardiac organoids as a model for drug screening to study cardiac dysfunction in the context of SARS-CoV-2 infection [115]. In their study, Mills et al. mimicked SARS-Cov-2 infection in cardiac organoids using different cytokines. These cytokines caused diastolic dysfunction similar to SARS-Cov-2 infected k18 hACE2 mice. A targeted drug screen identified bromodomain and extra-terminal family (BET) inhibitors as potential therapeutic agents. Two BD2-selective BET inhibitors, RXV-2157 and apabetalone, were also effective against COVID-19 damage.

From 1953 to 2013, 14% of drugs were withdrawn from the market due to their adverse cardiovascular effects [123]. Using iPSCs to evaluate drug cardiotoxicity can be vital in ensuring patient safety and helping healthcare providers select the right medications for their patients. The long-term use of chloroquine and hydroxychloroquine (HCQ) has been linked to a number of cardiac complications, according to a recent study by Kamp et al. [124]. The findings of their review have raised concerns regarding these drugs’ potential cardiotoxicity when treating SARS-CoV-2. Additionally, HCQ has been associated with the development of several cardiac arrhythmias, such as torsade de points, thereby favoring other medications for the treatment of SARS-CoV-2. In another study, Richards et al. worked on modeling pre-existing cardiovascular diseases, such as IHD, to study doxorubicin-induced cardiotoxicity [116]. Their study showed that doxorubicin decreased the amplitude of cardiac contractions and increased muscle actin disorganization. Additionally, Sallam et al. recently utilized an hPSC-derived cardiac organoid model to examine the cardiovascular impacts of tacrolimus and sirolimus, two immunosuppressant medications [117]. Sirolimus was found to limit fibrosis in cardiac clusters, indicating its possible role in limiting remodeling in patients with congestive heart failure.

HiPSC-derived and organoid-based systems offer powerful platforms for disease modeling and high-throughput drug screening; however, their application in toxicological testing remains limited. Traditional toxicology assays, such as LDH release, ATP-based viability, and electrophysiological readouts (e.g., MEA for cardiomyocytes), have been adapted for use in these models; however, standardization across laboratories is still evolving. The predictive value of these in vitro models compared to in vivo systems remains an area of active research, especially for systemic and chronic toxicity. Furthermore, current models often lack vasculature, immune system components, and metabolic competence, limiting their ability to fully mimic human drug responses. Therefore, while promising for early phase screening, further validation and integration with complementary models are essential to ensure accurate safety profiling and successful clinical translation [70,125].

## 6. Therapeutic Applications and Bioengineering Approaches

Regenerative medicine is a field that deals with replacing or rejuvenating tissues and organs that have been damaged due to injury, aging, or other factors. With the rise of tissue engineering and cellular therapies, the field has had many applications, such as in Type 1 Diabetes Mellitus, skin wound healing, organ transplants, and other uses [126]. Since their discovery, organoids gained a lot of interest among researchers for various reasons. As for cardiac organoids, there is little research available, attributed to the complexity of cardiomyocytes. However, the expectations are toward being able to regenerate damaged hearts and restore functionality after injury. Gao et al. were successfully able to implant human cardiac muscle patches into pigs with myocardial infarction [127]. Human-induced pluripotent stem cells (hiPSCs) were used to create the patches. These cells were first reprogrammed from human cardiac fibroblasts and subsequently underwent differentiation into smooth muscle, endothelial, and cardiomyocytes. The patches that were beaten within 1 day of fabrication showed further signs of maturation after 7 days of dynamic culturing. Transplantation of cardiac patches resulted in decreased infarct size and wall stress, limited cardiac remodeling, and enhanced cardiac function.

Several methods have been investigated to boost cardiac repair and regeneration using cardiac organoids [128]. In a study on the ability of cardiac progenitor cells produced from human embryonic stem cells (hESC-CPCs) to mature into cardiomyocytes, Varzideh et al. found that transplantation of cardiac organoids into the peritoneal cavity of mice caused a more advanced level of maturation [129]. This is most likely because the peritoneal cavity provides a normal physiological environment, contrary to other methods. It also resulted in increased neovascularization compared to that in traditionally cultured cardiomyocytes. In another study, Tan et al. developed a scaffold-free cardiac organoid and enhanced it with electrically conductive nanowires (e-SiNWs) [130]. The contractile properties and scaffold conductivity of engineered cardiomyocytes were enhanced by the application of e-SiNWs. The organoid was composed of stromal cells, endothelial cells, human cardiac fibroblasts (hcFBs), and cardiomyocytes produced from human pluripotent stem cells (hPSC-CMs). In their study on rats with infarcted hearts, they found that nano-wired organoids administration resulted in faster contractile development of engrafted hPSC-CMs, graft vascularization, and remarkable recovery of cardiac function after exposure to ischemia and reperfusion injury. Moreover, nano-wired organoids reduced cardiac fibrosis and left ventricular wall thinning. In a different study, researchers were able to create an organoid reconfigured from 2D-to-3D during organogenesis, in which a mesh nano-electronic was incorporated into the system. Named a “cyborg”, the cardiac organoid developed had a great amount of resemblance to conventional hydrogel-based cardiac organoids in terms of the markers of mature cardiac organoids, including actin, α-actinin, and troponin T (TNT) [131].

## 7. Current Challenges and Perspectives

Cardiac tissue engineering has spearheaded an innovative approach to treating heart failure, major obstacles to clinical implementation persist. Critical barriers in cardiac tissue engineering include cell supply and viability, scaffold architecture, vascularization and seamless integration, electrophysiological maturation, preclinical and clinical validation, and challenges related to personalized therapies and cost efficiency. Various methods have been explored to address these issues. For example, scaffold-based materials are being developed to enhance vascular integration, and immunomodulatory biomaterials and patient-derived iPSCs can help minimize immune rejection. However, challenges persist, including limited cell viability, uniform tissue maturation, and cost-effective biomanufacturing of functional cardiac cells. Additionally, concerns regarding the use of embryonic stem cells due to ethical and immunogenicity issues remain, while adult stem cells exhibit limited regenerative potential. Developing scaffolds that replicate the complex architecture and mechanical properties of heart tissue is a major challenge. Achieving regulatory approval for bioengineered cardiac therapies, such as those from the U.S. FDA is crucial but is complicated by safety and immunogenicity concerns. Therefore, it is important to design biomimetic frameworks that closely resemble the natural qualities of heart tissue.

Cutting-edge technologies, including 3D bioprinting using bioinks with cells and growth factors, facilitate the creation of sophisticated and detailed heart structures. Additionally, organoid-on-chip systems have the potential to integrate cardiac organoids with other organ models, thereby creating dynamic multi-organ platforms for disease modeling. Although there have been several promising advancements in cardiac tissue engineering, cost-effectiveness and accessibility remain significant challenges due to the high costs of biomaterials and bioreactors. Advancements in 3D bioprinting, off-the-shelf biomaterials, and minimally invasive artificial tissue implants are crucial for regulatory approval and the future of cardiac repair and regeneration. Ultimately, developing patient-specific engineered tissues using their own cells remains the ultimate goal, but overcoming technical complexities and cost barriers is essential to revolutionize heart failure treatment and improve patient outcomes.

## 8. Conclusions

Bioengineering plays a pivotal role in treating heart failure by enabling the repair of damaged cardiac tissue, potentially eliminating the need for heart transplantation. Significant breakthroughs have been made in engineering functional cardiac tissues, driving progress in regenerative therapies. By integrating sensors for early diagnosis and real-time monitoring, bioengineering bridges the critical gaps in cardiac research and clinical care. Tissue engineering, cell-based regenerative therapy, and 3D bioprinting are advancing the development of physiologically relevant cardiac tissue models. This interdisciplinary field synergizes stem cell biology, bioprocessing, advanced biofabrication, and materials science to accelerate the development of cardiac tissue. With its potential to deliver personalized, regenerative, and minimally invasive therapies, bioengineering could revolutionize cardiology by reducing dependence on transplantation and lifelong medication. Recent breakthroughs in stem cell therapy and bioengineered heart tissues have shifted the treatment paradigm from reactive to predictive and preventive care. The integration of smart bioelectronics and computational modeling promises to further transform cardiac disease management, making therapies more precise, accessible, and patient-specific. However, despite remarkable progress in heart disease modeling, the inherent complexity of cardiac tissue poses challenges in fully replicating its intricate structure and pathophysiology. To overcome these challenges and improve clinical translation, future cardiac tissue engineering iterations should incorporate immunomodulatory scaffold designs that enhance biocompatibility and integration. This can be achieved through close collaboration among engineers, biologists, and clinicians. By combining expertise in biomaterials, stem cell science, and clinical medicine, functional heart tissues can be developed for next-generation regenerative therapies.

## Figures and Tables

**Table 1:** This table lists the different iPSC-derived cardiac disease models and tools for drug screening and congenital heart disease modeling, along with their validation phases and performance results.

Device/Model & Author	Specific Application	Stage of Validation	Data Variability & Performance Metrics
iPSC-derived Cardiomyocytes [92]	Modeling Hypoplastic Left Heart Syndrome (HLHS)	In vitro	Moderate reproducibility; downregulation of MESP1, TNNT2, GJA1; delayed GATA4 expression. Functional immaturity and altered calcium handling observed across patient-specific lines.
Cardiac Progenitor Cells [91]	Studying genetic disruption in early cardiogenesis (HLHS)	In vitro	Consistent gene expression changes across HLHS samples; impaired growth factor response; premature cell cycle exit and apoptosis uniformly observed.
TOF-specific Cardiomyocytes [94]	Modeling Tetralogy of Fallot (TOF) and identifying causal mutations	In vitro	Low interline variability; reproducible effects of TP53, FBLN2, DAAM2 mutations on myocardial maturation and WNT signaling. Robust gene-disease correlation.
TOF-specific iPSCs [97]	Exploring TBX1 mutation effects on neural crest migration in TOF	In vitro	The YAHKMUi001-A cell line can be used as a tool for in vitro modeling of TOF for etiology research or gene therapy.

**Table 2:** This table summarizes the various iPSC-derived cardiac disease models and devices used for ischemic heart disease modeling and drug screening, along with their validation stages and performance outcomes. Abbreviations: iPSC, Induced pluripotent stem cell; CM: Cardiomyocyte; IHD: Ischemic heart disease; DMD, Duchenne muscular dystrophy; MAP4K4: Mitogen-Activated Protein Kinase Kinase Kinase Kinase 4; BET: Bromodomain and Extra-Terminal domain; hPSC, Human pluripotent stem cell.

Device Name/Model	Specific Application	Stage of Validation	Data Variability and Performance Metrics
3D Cardiac Organoids [10]	Modeling myocardial infarction under hypoxic conditions	In vitro	Mimicked infarction features: fibrosis, altered calcium handling
hiPSC-CMs under hypoxia [105]	IHD modeling and levosimendan drug testing	In vitro	Hypoxia slowed Ca²⁺ transients, caused sarcomere disarray; drug showed an antiarrhythmic effect
Engineered Cardiac Patches [108,109,110,111]	Myocardial regeneration after ischemia (fibrin/collagen hydrogels, cell sheets, bioprinted structures)	Preclinical/in vivo	Structural integration in animal models; clinical trials underway in some cases
iPSC-CMs [112]	DMD cardiomyopathy modeling and CRISPR correction	In vitro	Restored dystrophin expression and improved contractility after 50% correction
3D DMD Cardiac Organoids [113]	Identification of dysregulated miRNAs in DMD	In vitro	Identified 5 miRNAs enriched in disease state; supports molecular mechanism exploration
iPSC-CMs + H_2_O_2_ injury [114]	Drug screening post-ischemia; MAP4K4 gene targeting	In vitro	Identified a small molecule increasing post-injury survival, validated via gene silencing
Human Cardiac Organoids [115]	COVID-19 cytokine storm modeling and drug screening	In vitro & animal model	Diastolic dysfunction replicated; BET inhibitors reversed damage
iPSC-CMs [116]	Cardiotoxicity testing of doxorubicin in the ischemic heart disease context	In vitro	Reduced contraction amplitude, actin disorganization
hPSC-derived Cardiac Organoids [117]	Cardiotoxicity evaluation of immunosuppressants (tacrolimus, sirolimus)	In vitro	Sirolimus reduced fibrosis in cardiac clusters; therapeutic potential for heart failure

## Abbreviations

The following abbreviations are used in this manuscript:

CHD	Congenital Heart Disease
iPSCs	Induced Pluripotent Stem Cells
BAVD	Bicuspid Aortic Valve Disease
CAVD	Calcific Aortic Valve Disease
HLHS	Hypoplastic Left Heart Syndrome
TOF	Tetralogy of Fallot
IHD	Ischemic Heart Disease
hiPSC-CMs	Human-Induced Pluripotent Stem Cell-Derived Cardiomyocytes
hPSC-CMs	Human Pluripotent Stem Cell-Derived Cardiomyocytes
MAP4K4	Mitogen-Activated Protein Kinase Kinase Kinase Kinase 4
SARS-CoV-2	Severe Acute Respiratory Syndrome Coronavirus 2
BET	Bromodomain and Extra-Terminal
DMD	Duchenne Muscular Dystrophy
miRNAs	MicroRNA
CRISPR/Cas9	Clustered Regularly Interspaced Short Palindromic Repeats/CRISPR-associated protein 9
e-SiNWs	Electrically Conductive Silicon Nanowires
hcFBs	Human Cardiac Fibroblasts
hESC-CPCs	Human Embryonic Stem Cell-Derived Cardiac Progenitor Cells
FDA	Food and Drug Administration
3D	Three-Dimension
